# Performance of solar roof top panels with disparate particulate accumulation: Exergy analysis on an indoor lab study

**DOI:** 10.1371/journal.pone.0291018

**Published:** 2023-09-05

**Authors:** Muhammad Alam Zaib Khan, Abdul Wahab, Fawad Ali, Naveed Ahmad, Muhammad Ali Kamran, Ali Hassan

**Affiliations:** 1 Mechanical Engineering Department, University of Engineering and Technology Peshawar, Peshawar, Pakistan; 2 Thermal Systems Engineering Department, U.S–Pak Center for Advanced Studies in Energy, University of Engineering and Technology Peshawar, Peshawar, Pakistan; 3 Mechanical Engineering Department, University of Engineering and Technology Taxila, Taxila, Pakistan; Southwest Jiaotong University, CHINA

## Abstract

Deployment of solar photovoltaic panels are significantly rising to tackle adverse effects of climate change however, factors affecting output need to be categorized in addition to latitude angle and space. It is important to consider the atmospheric impact which can drastically change output power of solar panels. This study covers dust accumulation of soil, sand and ash at variable weights to foresee its effects on panel power output. Mixtures of these particles at multiple constituents were also analyzed. Experimental results indicated that clean panel gives maximum power output of 21.37W and exergy efficiency of 7.96% whereas ash accumulation showed worst results of 2.88W power output and 1.07% exergy efficiency at 700W/m2 and 50g dust accumulation. Other parameters like energy destruction, exergy losses and sustainability index were also analyzed. Trends have been illustrated in graphs along with the change in solar intensity and dust accumulations.

## 1. Introduction

The agenda of 2030 for Sustainable Development highlights multiple interrelated goals which also includes clean energy and climate change. To acknowledge the importance of these goals, efforts are being applied to push the governments and academia to conduct more research for successful implementation of these goals. SDG-7 represents clean and affordable energy which is considered in this study [1]. Thus, renewable energy technologies are the major sources for energy transition as it can supply two third of the total energy and can cut the greenhouse gas emissions needed to achieve 2050 targets [2]. Climate change is the largest threat for the societies in future therefore, clean energy is the only way to mitigate the climate change. SDG-13 indicates climate action which needs to be taken into account to promote well-being [3]. Over decades, climate change has been a main concern for the future of living beings and earth. Fundamental cause of global warming is the immense fossil fuel use in generation of power for industries, household activities and for transportations. Research on large scale has been carried out to mitigate use of fossil fuel by replacing them with alternate energy resources [4]. In research, it was suggested that CO2 emissions can be reduced around the world if governments decrease the subsidies on fossil fuels by 20 cents [5]. But clearly, this is not an ideal solution due to growing energy demand all over the world. Other than fossil fuels, there are two more resources that can generate energy on a large scale with minimum greenhouse gases emissions, nuclear and renewable energy [6]. Power generated by renewable energy resources is most sustainable with lowest emissions [7, 8].

Solar PV is one of the renewable energy technologies that can accelerated for high growth of energy around the globe. However, efficiency of solar PV is critical and factors affecting it must be studied carefully to eradicate them. One of the cases is the dust accumulation over PV surface [9]. Solar energy is available abundantly and can be utilized for thermal and electrical power generation [10]. Immense amount of energy is emitted by the sun in form of electromagnetic radiations and only 1336 W/m2 is reached at the earth atmosphere (100 kms above the sea level) and it reduced to around 1000 W/m^2^ when reached earth surface [11]. Amount of energy reached earth’s surface can be directly harnessed for electrical energy using photovoltaics or thermal energy using solar collectors [12]. Photovoltaics has gained huge importance in reducing use of fossil fuels due to solar radiation conversion to electrical power but some absorbed energy by photovoltaics is used by electrons for heat generation instead of converting it to electrical energy, and some amount of solar energy is also wasted due to dust present on the photovoltaics surface [13, 14]. Heat generation in electrons cause reduction in electrical power production.

To minimize the surface temperature caused by heat generation of electrons, active and passive cooling techniques can be applied. Use of nanofluids, water and oils are included in active cooling of photovoltaics while attaching fins on back of PV, using fan and PCM are passive cooling techniques for PV. A comprehensive review was completed on performance of nanofluid in thermal collectors, photovoltaics, solar stills, thermal energy storage and solar pond [15]. Hassan et al. [16] used graphene/water nanofluid and achieved 23.9 oC decrease in PV surface temperature with maximum enhancement of 23.9% in electrical efficiency. Wahab et al. [17] achieved electrical exergy efficiency of 13.02% using RT-35HC PCM with graphene/water nanofluid.

Besides increment in PV surface temperature, PV performance is also highly affected by environmental conditions. Some radiation is absorbed by the dust particles available on the PV surface and also in the atmosphere. These dust particles caused optical loss resulting in reduced irradiance on PV cells, ultimately leading to reduced performance of PV [14]. Various forces (van-der Waals force, electrostatic force, gravity and capillary force) caused dust particle adhesion on the PV surface, depending on particle size, properties of material and distance between particle and surface [18]. Lorenz-Mie scattering theory described the sunlight absorption and scattering owing to dust particles available on PV surface, this scattering of sunlight caused decrement in output power of PV module [19, 20]. El-Shobokshy and Hussein et al. [21] showed that output power, short-circuit current, fill factor and decrement in solar intensity as a function of dust particle deposition density by performing a laboratory experiment. Garg et al. [22] found 8% average reduction in transmittance after placing the glass at 45 deg tilt angle for 10 days exposure period in Roorkee, India. Salim et al. [23] studied the effect of dust deposition on a PV array near Riyadh, Saudi Arabia, energy output was reduced by 32% in 8 months due accumulation of dust particles on PV surface. Dust deposition on PV is affected by changing the tilt angle. Elminir et al. [24] found that densities of dust deposition varies from 15. 84 to 4.48 g/m^2^ when tilt angle was increased from 0 to 90o. Dust accumulation on PV surface caused reduced solar energy absorption by the PV surface. It causes reduction in transmittance of solar radiations over PV cells, leading to low power output. However, dust accumulation experimentation was carried out by Katoch et al. [25]. To compare the sand dust on PV surface and its impact compared to clean panel. The average power loss was 24% with 10.5 g/m2 of dust. It was suggested that more concentration is needed to study the effect of dust over PV surface. However, study of sand is not enough as there are other dust particles in the atmosphere as well which keeps this study very limited for real case scenario. Fan et al. [26] developed the dust concentration and energy conversion efficiency (DC-ECE) model for PV. It was found that conversion efficiency of PV was reduced by raising the dust particle size.

A substantial role has been played by exergy analysis to improve PV performance. During an investigation by Joshi et al. [27], energy and exergy efficiencies were compared for PV/T system with two different realistic methods and it was depicted that exergy analysis provided better outcomes. Said et al. [28] reported that 40–80% air humidity can enhance the adhesion rate of dust to 80%. Different studies found that dust can reduce the PV performance by 2–10% [29–31]. Similarly, Pathak et al. [32] performed exergy analysis to compare PV modules performance. In order to select PV system for roof top, exergy analysis was performed for better implementation. Hence, exergy analysis has vital importance when it comes to practical implementation. Hachicha et al. [33] reported that dust accumulation decreases with increase in tilt angle of PV. The daily average efficiency of PV was reduced by 58.2% due to enhanced dust accumulation by reducing the tilt angle from 45° to 0° [34]. The performance of PV panel is also dependent on the type of dust particles. Chanchangi et al. [35] found that ash dust particles caused high power deterioration by up to 98% compared to bird droppings, carpet dust, clay, salt, sandy soil and wood dust. The soiling of PV module raised the cost of power generation. However, this study was limited to each type of dust impact separately but in reality, all these dusts are present in the atmosphere so mixture of dust needs more attention to make experimentation close to real time scenario. Shahabaddin et al. [36] predicted the increment in power production cost by 100–270 $/MWh due to dust accumulation. Therefore, it is essential to investigate the irreversibility generated to deteriorate the PV performance due to soiling effect. The second law of thermodynamics or exergetic study can provide a detailed analysis of irreversibities that can be generated during dust accumulation. To overcome, the limitation of this study, the present research conducted fully focuses on the exergy analysis to deal with the irreversibilities caused due to dust accumulation. Jathar et al. [37] provided a comprehensive review of the environmental aspects on PV systems performance. The study also considered the advanced steps that can help reduce the negative impacts on PV output that leads to the deterioration of PV performance. Useful results extracted from recent research involve PV performance with respect to the degree of deviation of sun, rise in temperature of PV panel, dust accumulation impact on PV panel, wind velocity impact, relative humidity, and shadow impacts. Khan et al. [38] considered the impact of tilt angle on PV performance. Advancements in sun tracking techniques have been proven quite effective and challenging for improving PV performance as tilt angle changes based on months, seasons, locations and orientations etc., thus finding optimum output in a single attempt seems inappropriate. Furthermore, the extra cost linked with sun tracking especially the initial, operating, and maintenance cost is higher. However, it enhances the output of PV performance to a great extent.

This study consists of an indoor experimentation on solar photovoltaic cells with variable dust particles accumulation over PV surface to investigate the impact of different dust particles on the output power. Considering the energy transition from fossil fuels to renewable energy, solar panels have been used as an off-gird system at mining industries and sites located far away from the national grids. Purpose of this research is to study the impact of atmospheric dust accumulation on photovoltaic panels installed at mining sites which drastically reduces the output power. The dust particles in the atmosphere at mining sites is usually a mixture of sand, soil and ash. No study has been conducted before considering the mixture of all these dust particles and its effect on the PV panel output which indicates a major research gap. In addition to this, exergy analysis to determine the detailed analysis of irreversibility generated during dust accumulation is not performed according to the knowledge of authors which also points a major research gap. Therefore, this study involves the experimentation of dust particle mixtures over PV surface to visualize its impact on power output and explains the exergy analysis for all specified proportions in terms of exergy output, exergy efficiency, exergy losses, exergy destruction, entropy generation and sustainability index which have not been mentioned in previous studies thus stating novelty and bridging knowledge gap. Moreover, this study will be helpful in creating critical awareness to the companies using solar panels as a renewable power production resource at mining sites.

## 2. Methodology

### 2.1 Experimental setup

One monocrystalline panel of Cells Germany was used with 30 W power capacity in this experimental investigation. Specifications of PV panels are highlighted in Table 1. Experiments were undertaken in a controlled environment. Solar panel module was non-tracking and fixed at the latitude angle of 34°.

**Table 1:** PV panel specifications.

Type	Mono
Model	ASL 30–12
Tolerance	±3%
V_nominal_	12V
P_max_	30W

Experiments were conducted in the laboratory at specified temperature and pressure with varying intensity and dust accumulation. Following PV panels were considered during experimentation:

1. **Case A:** Clean PV panel
2. **Case B:** PV panel with sand accumulation
3. **Case C:** PV panel with soil accumulation
4. **Case D:** PV panel with ash accumulation
5. **Case E:** PV panel with mixture (sand as main constituent) accumulation
6. **Case F:** PV panel with mixture (soil as main constituent) accumulation
7. **Case G:** PV panel with mixture (ash as main constituent) accumulation
8. **Case H:** PV panel with mixture (all constituents in equality) accumulation

**Fig 1:**
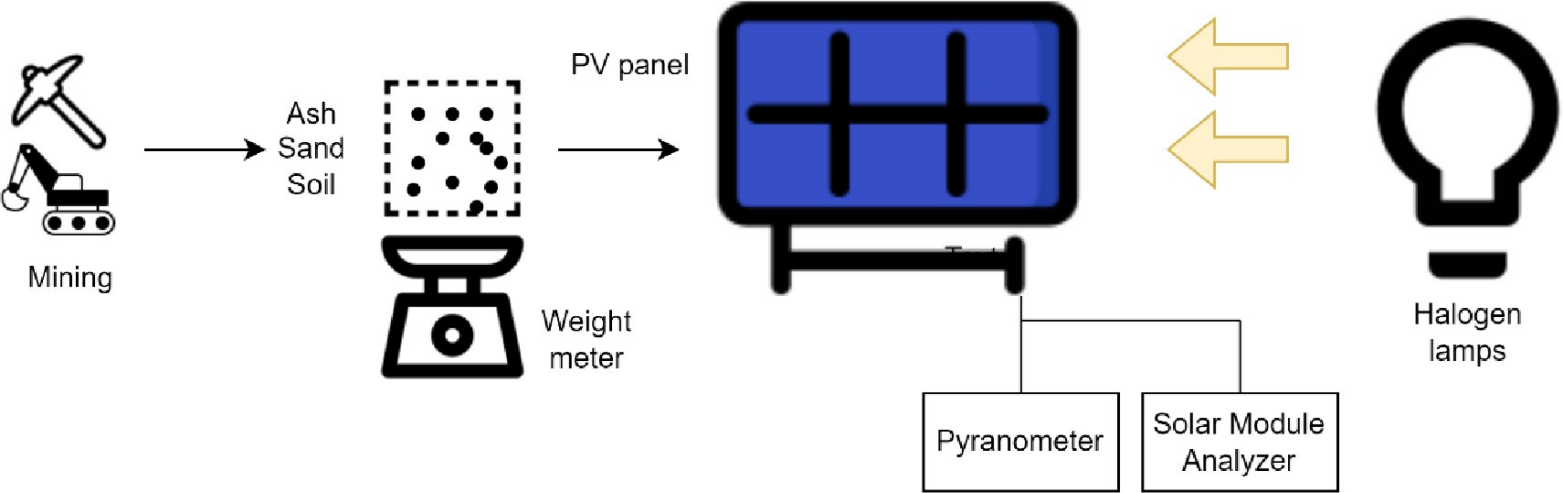
Schematic diagram of test bench.

**Fig 2:**
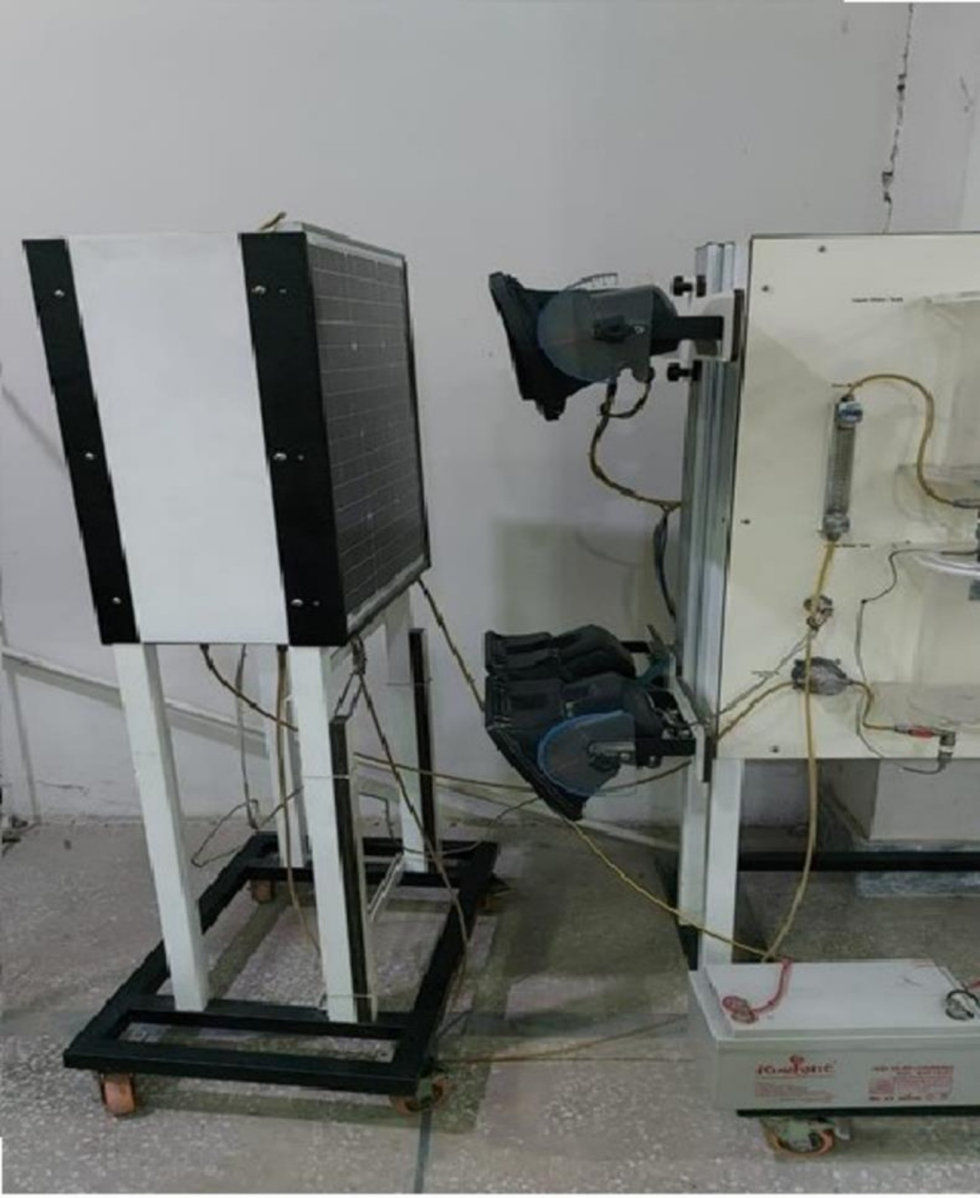
Experimental setup of test bench.

Fig 1 shows the schematic diagram of the test bunch and Fig 2 shows the experimental setup which was used for an indoor study of dust accumulation on photovoltaic panels at 30g and 50g and variable light intensity 500–700 W/m^2^ in controlled conditions. Latitude angle of Peshawar was considered as 34° for experimentation considering the real conditions.

**Fig 3:**
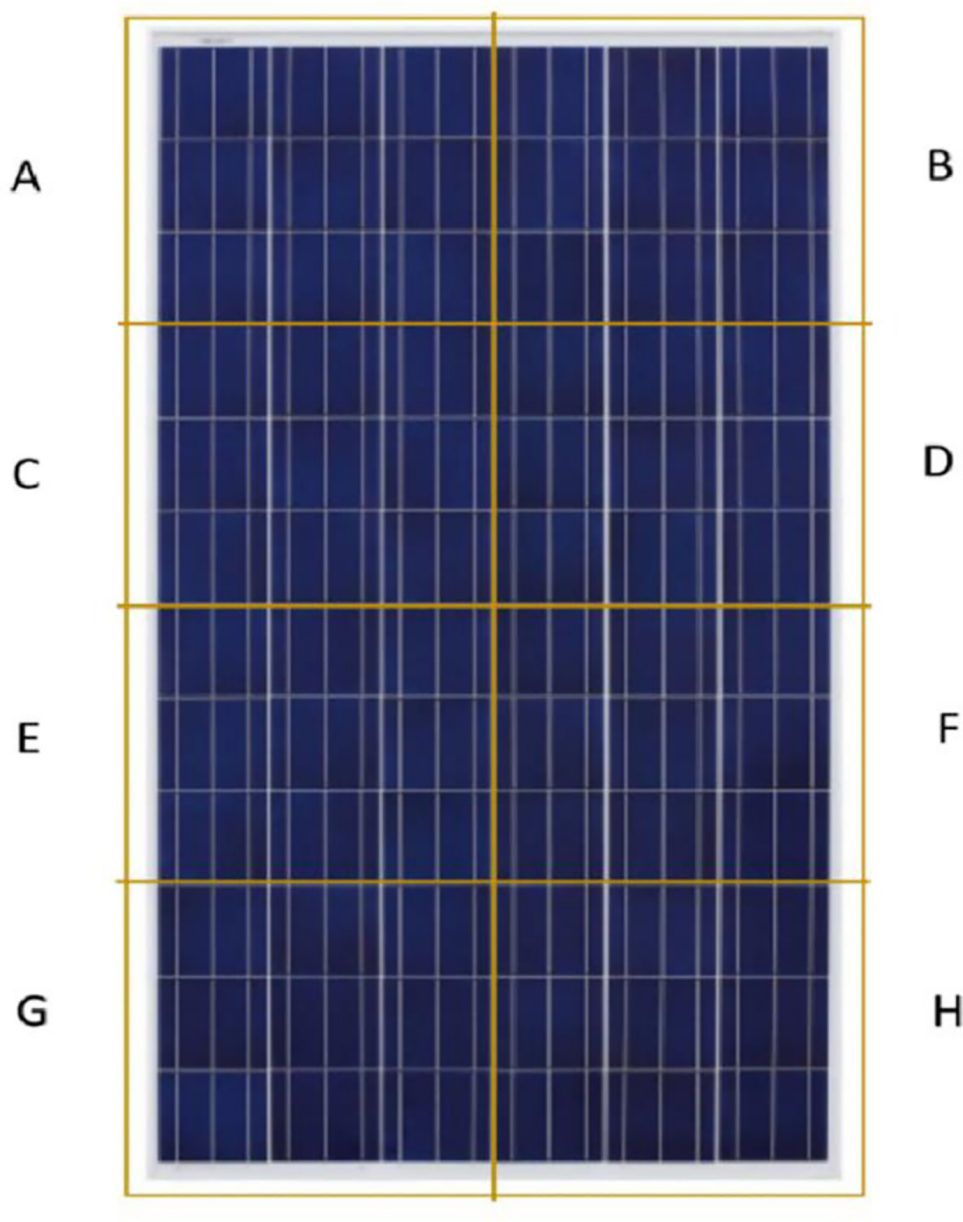
PV panel segments for evenly dust distribution.

PV panel was divided into 8 small portions named as A, B, C, D, E, F, G and H as shown in Fig 3. The overall dust was divided into 8 parts for each segment and was uniformly spread in each segment to make sure the dust is uniformly spread over the PV panel surface. Length of the panel was divided into 4 equal parts and width of the panel was divided into 2 equal parts. Points were marked to make sure each section is treated separately for dust segregation. In this study, length of PV module was 680mm and width was 300mm therefore, each segment’s dimensions were 170mm length and 150mm width.

This study focused on a dust thickness of 100 microns, which is the maximum particle size that air carries in residential and domestic areas. The assumption was that the same dust particles found in mining areas would also agglomerate on nearby PV panels. As a result, dust samples with a maximum particle size of 100 microns were obtained from the local market for use in the experiments. A rectangular frame mold was used with a suitable height based on each case to make sure that dust is uniformly distributed over the PV surface. The thickness of dust for each case varied so the rectangular frame helped to maintain the surface’s uniform with respect to the specific case.

The test was initiated by setting up the test bench, which involved fixing halogen lights in front of the solar panel to ensure that the rays hit the panel surface at a 90-degree angle. After arranging the halogen lights, the light intensity was fixed using a pyranometer device. As this study involved multiple intensity levels, all the intensity variation checkpoints were set up on the test bench. The power wires of the PV module were connected with the Solar Module Analyzer to record the readings, which represented the power output of the PV panel. The PV panel surface was divided into eight segments to ensure uniform dust accumulation. Dust particles were segregated using a mesh to ensure that only particles under 100 microns in size were used in this experimental study. These segregated particles were weighed using a precise weight machine and then divided equally into eight segments, which were then spread uniformly on the surface of the PV panel. Variation in dust accumulation as mentioned in cases was studied to analyze the effect of different types of dust (ash, soil, and sand) on the PV panel. Mixtures of dusts were also studied with different constituents to mimic actual outdoor working environments in commercial and residential areas. Dust accumulation considered in this study was the amount of dust accumulated on the PV surface at mining sites in real-time cases on which the wind velocity already had a certain impact. So, this study focused on that accumulated amount of dust on the PV surface which neglects the requirement of wind velocity during the experimentation in the lab.

### 2.2 Measuring instruments

Instruments used for measurements are enlisted below:

- Solar Module Analyzer for measuring the PV power. The model used was PROVA 210, MTAM-00111 from TES product
- Pyranometer for measuring solar intensity on PV surface
- Digital weight meter
- Halogen lights

#### 2.2.1 Pyranometer

It is used to measure solar intensity over PV surface to the entire amount of sunlight getting on a horizontal plane. We needed a pyranometer to measure the intensity of light that was reaching our panel so that we could adjust it according to our need. The pyranometer used had a 4-digit LCD display with 2000W/m^2^ range and 0.1W/m^2^ resolution. Characteristics of the pyranometer are shown in Table 2.

**Table 2:** Pyranometric specifications.

Specification	Description
Range	2000W/m^2^
Resolution	0.1W/m^2^
Spectral response	400nm-1100nm
Accuracy	Typically, within ±10 W/m^2^
Angular accuracy	Cosine Corrected
Calibration	User recalibration available
Model	TENMARS TM-207

#### 2.2.2 Digital weight scale

Digital weight scale was used to find out the weight of pollutant before spreading it on panel. A fixed amount of dust was to be placed on panel to check its effect on the panel’s output. Therefore, the digital weight scale was equipped with a high precision “strain-gauge” sensor.

#### 2.2.3 Halogen lights

**Fig 4:**
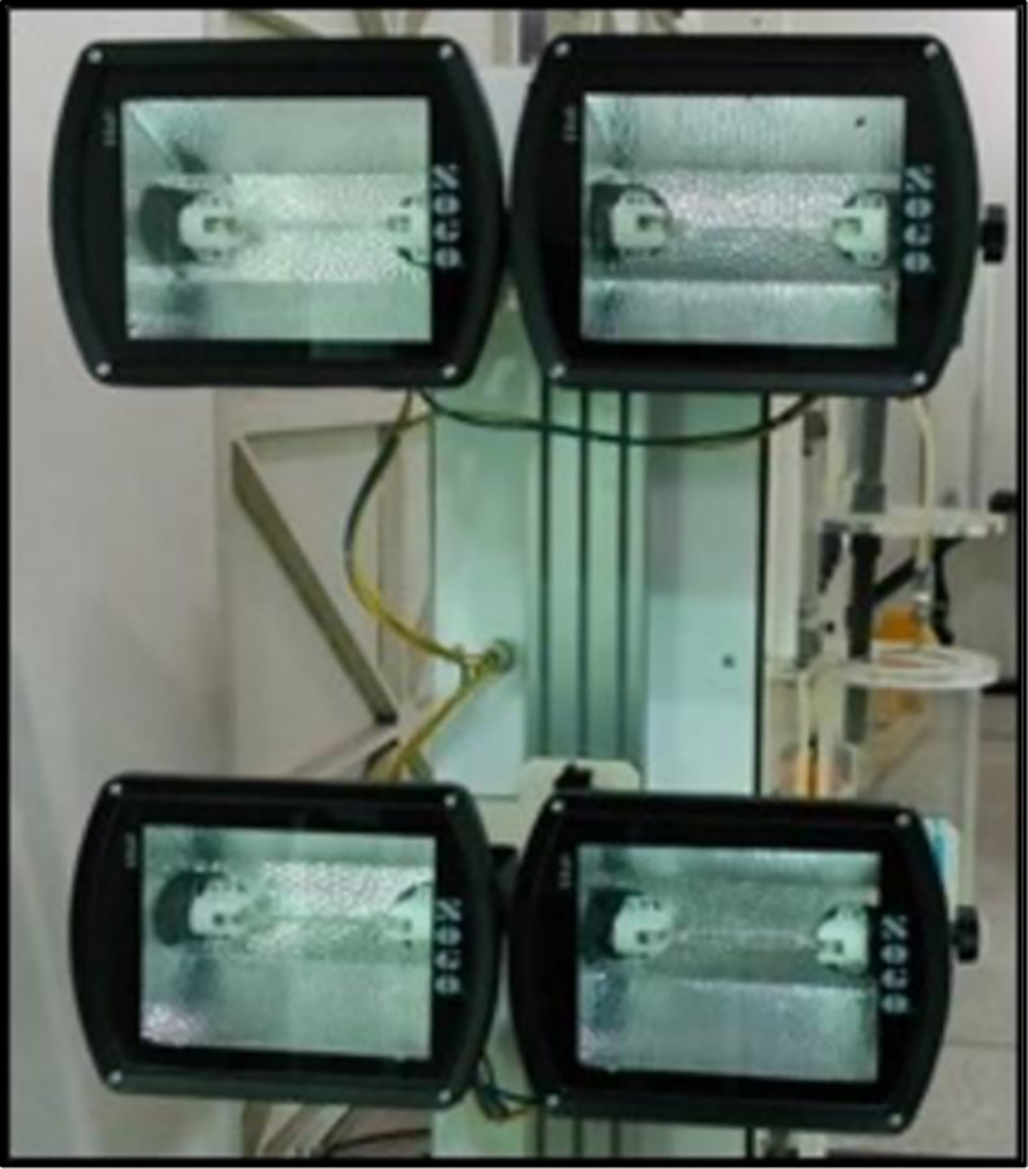
Halogen light used during indoor experiments.

Halogen lights were the main component of our experimental setup. “Phillips bulb each of 500W was used for this experimentation. The tungsten halogen lamp was widely used in solar simulators because it delivers a very smooth and stable spectral output. The wavelength is between 360–2500 nm, which is similar to sunlight, particularly in relation to thermal radiation. Fig 4, shows the lights which were used in this experimental study.

### 2.3 Formulas for data interpretation

Energy and exergy efficiencies were calculated using following formulas [39]:

$${\dot{G}}_{eff}=I_{s}*A_{panel}$$ (1)

where ‘*A*_*panel*_’ represents the area of the PV panel and ‘*I*_*s*_’ is the solar intensity. Hence, electrical efficiency (*η*_*elect*_) of PV panel was calculated by [39]:

$$\eta_{elect}=\frac{P_{max}}{{\dot{G}}_{eff}}$$ (2)

where ‘*η*_*elect*_’ represents electrical efficiency and ‘*P*_*max*_’ is maximum power.

The steady state exergy balance of the system is as follows [40]:

$${Ėx}_{in}={Ėx}_{out}+{Ėx}_{dest}+{Ėx}_{loss}$$ (3)

where ‘${Ėx}_{in}$’ is the exergy input rate, ‘${Ėx}_{out}$’ is the exergy output rate and ‘${Ėx}_{dest}$’ is the exergy destruction rate of the system.

Steady state exergy equation is as follows [40]:

$${Ėx}_{in}+{Ėx}_{sun,in}={Ėx}_{out}+{Ėx}_{dest}+{Ėx}_{loss}$$ (4)

where ‘${Ėx}_{in}$’ is the exergy input rate, ‘${Ėx}_{out}$’ is the exergy output rate, ‘${Ėx}_{loss}$’ is the exergy loss and ‘${Ėx}_{dest}$’ is the rate of exergy destruction. Thus, exergy loss (${Ėx}_{loss}$) is [40]:

$${Ėx}_{loss}={Ėx}_{loss,conv}+{Ėx}_{loss,rad}$$ (5)

where ‘${Ėx}_{loss,conv}$’ is convection exergy loss and ‘${Ėx}_{loss,rad}$’ is radiation exergy loss and can be measured as [40]:

$$\{{Ėx}_{loss,conv}={\dot{Q}}_{loss,conv}\left(1-\frac{T_{0}}{T_{surf}}\right)=hA\left(1-\frac{T_{0}}{T_{surf}}\right){Ėx}_{loss,rad}={\dot{Q}}_{loss,rad}\left(1-\frac{T_{0}}{T_{surf}}\right)==\varepsilon \sigma A(T_{surf}^{4}-T_{0}^{4})\left(1-\frac{T_{0}}{T_{surf}}\right)$$ (6)

where ‘${\dot{Q}}_{loss,conv}$’ is convection energy loss, ‘${\dot{Q}}_{loss,rad}$’ is radiation energy loss, ‘*h*’ is convective heat transfer coefficient, ‘*σ*’ is Stefan’s Boltzmann constant and ‘*ε*’ is emissivity. Solar exergy input rate ‘${Ėx}_{solar,in}$’ can be determined by [40]:

$${{Ėx}_{in}=Ėx}_{solar,in}={Ėn}_{in}[1+\frac{1}{3}{\left(\frac{T_{0}}{T_{sun}}\right)}^{4}-\frac{4}{3}(\frac{T_{0}}{T_{sun}})]$$ (7)

where ‘*T*_0_’ is the ambient temperature and ‘*T*_*sun*_’ is sun temperature i.e., 5800 K. Thus, rate of exergy output (${Ėx}_{out}$) equal to overall exergy (${Ėx}_{overall}$) is determined as [40]:

$${Ėx}_{out}={Ėx}_{overall}={Ėx}_{elect}$$ (8)

where ‘${Ėx}_{elect}$’ is the electrical exergy rate. Thus, electrical exergy rate will be equal to:

$${Ėx}_{elect}={Ėn}_{elect}$$ (9)

where ‘${Ėx}_{elect}$’ is electrical exergy rate and ‘${Ėn}_{elect}$’ is electrical output rate [40].

$$\psi_{elect}=\frac{{Ėx}_{elect}}{{Ėx}_{solar,in}}$$ (10)

Hence, overall exergy efficiency (*ψ*_*overall*_) will be equal to [40]:

$$\psi_{overall}=\psi_{elect}$$ (11)

Furthermore, entropy generation rate (${\dot{S}}_{gen}$) can be defined by [40]:

$${\dot{S}}_{gen}=\frac{{Ėx}_{dest}}{T_{0}}$$ (12)

Sustainability index is as follows [40]:

$$SI=\frac{1}{1-\psi_{PV}}I\dot{P}=(1-\psi ){Ėx}_{loss}$$ (13)

where *ψ* is the system’s overall exergy.

### 2.4 Uncertainty analysis

Uncertainty analysis was performed as per method described by [41] was used to calculate the uncertainty in the experimental study results. Uncertainty analysis shows the maximum possible deviation of measured results. Thus,

$$R=f(v_{1},v_{2},\ldots .,v_{n})$$ (14)

Function uncertainty *R* can be measured as follows:

$$\delta R=\sqrt{{\left(\frac{\partial R}{{\partial v}_{1}}\delta v_{1}\right)}^{2}+{\left(\frac{\partial R}{\partial v_{2}}\delta v_{2}\right)}^{2}+\cdots +{\left(\frac{\partial R}{\partial v_{n}}\delta v_{n}\right)}^{2}}$$ (15)

$$\frac{\delta R}{R}=\pm \sqrt{{\left(\frac{\delta v_{1}}{v_{1}}\right)}^{2}+{\left(\frac{\delta v_{2}}{v_{2}}\right)}^{2}+\cdots +{\left(\frac{\delta v_{n}}{v_{n}}\right)}^{2}}$$ (16)

Considering Eq 16, electrical efficiency uncertainty will be written as:

$$\frac{{\delta \eta }_{el}}{\eta_{el}}=\pm \sqrt{{\left(\frac{\delta P_{m}}{P_{m}}\right)}^{2}+{\left(\frac{\delta {\dot{G}}_{eff}}{{\dot{G}}_{eff}}\right)}^{2}}$$ (17)

Similarly, electrical exergy efficiency uncertainty will be:

$$\frac{{\delta \psi }_{el}}{\psi_{el}}=\pm \sqrt{{\left(\frac{\delta \dot{{Ex}_{el}}}{\dot{{Ex}_{el}}}\right)}^{2}+{\left(\frac{\delta {\dot{E}}_{in}}{{\dot{E}}_{in}}\right)}^{2}}$$ (18)

The uncertainty result is mentioned in Table 3.

**Table 3:** Uncertainty of parameters and efficiencies.

Parameter and properties	Accuracy	Uncertainty
Pyranometer	±1.0	0.11%
Maximum Power	±0.1	0.43%
Electrical Efficiency	-	±0.44%
Electrical Exergy Efficiency	-	±1.25%

## 3. Results and discussion

This study was carried out in a controlled environment to better understand the effect of light intensity and dust accumulation on solar PV panels. Light intensity was varied from 500 W/m^2^ to 700 W/m^2^. For characterizing the effect of dust accumulation i.e., sand, soil and ash were used during experimentation. The results achieved from Case “A”, “B”, “C”, “D”, “E”, “F”, “G” and “H” were further analyzed with varying intensity and dust factor. This study focused on two weight parameters and 8 cases for various types of dust accumulations. All these cases of dust accumulations show different behavior to incoming light radiations which vary the output power of the PV panel. Furthermore, the change in intensity will have certain effects on the output power with respect to each dust accumulation case in this study. This experimentation was performed considering the environmental conditions of Peshawar, Pakistan.

### 3.1 Exergy output

**Fig 5:**
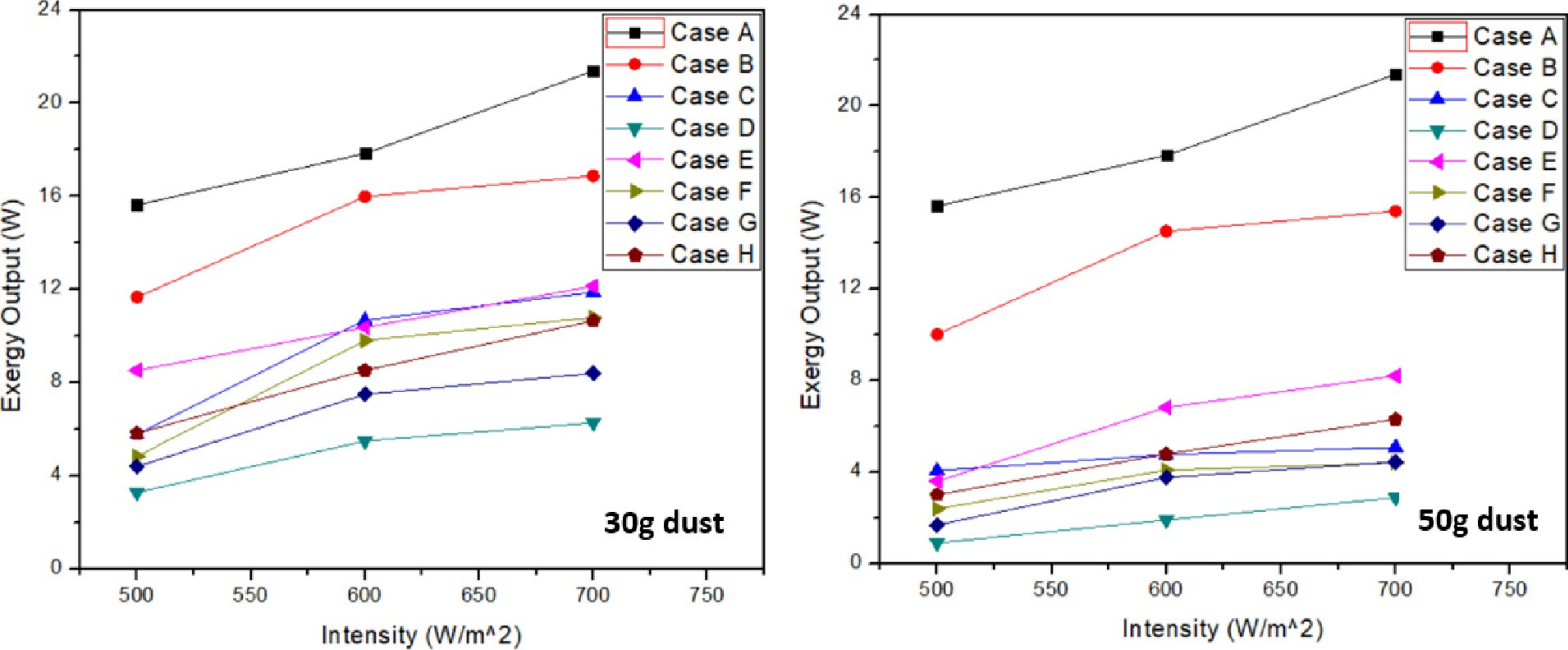
Exergy output/ electrical output at 30g and 50g dust accumulation (A: Clean PV; B: PV with sand accumulation; C: PV with soil accumulation; D: PV ash accumulation; E: PV with mixture (sand as main constituent) accumulation; F: PV with mixture (soil as main constituent) accumulation; G: PV with mixture (ash as main constituent) accumulation; H: PV with mixture (all constituents in equality) accumulation).

In order to get actual energy available, exergy analysis was performed after experimentation for PV systems. Electrical output is equal to exergy output in this study as there is no thermal energy involved. Furthermore, electrical exergy was basically the total exergy output in this experimentation. Fig 5 shows the graphs at 30g and 50g dust accumulation with varying light intensity from 500–700 W/m^2^. It can be seen from the graph that clean panel (case A) output is more than all other cases at all light intensities. Thus, it was concluded that dust accumulation results in the decrease of electrical power. Lowest electrical output was achieved with ash accumulation. Effect of sand accumulation on PV panel output was less compared to soil and ash accumulation. Similarly, considering the mixture of all dust accumulation in which sand was a major constituent, output of PV panel was more as compared to soil and ash as major constituents.

From the Fig 5 below, it can be seen that foreign particles on solar panel directly effects the output of electric power. Furthermore, as the foreign particles accumulation on solar panel increases, power output simultaneously reduces. At 700 W/m^2^ intensity and 30g dust, decrease in exergy output with sand, soil and ash was 21.05%, 44.42% and 70.67% respectively when compared with clean panel. Similarly, at 50g dust, decrease in exergy output with sand (case B), soil (case C) and ash (case D) dust particles was 27.95%, 76.26% and 86.53% respectively when compared with clean panel. In both cases, ash dust particles constitute maximum power deterioration compared to sand and soil dust particles. It was depicted that tiny particles of dust prevent the solar radiation to pass through leading to output power reduction. However, it was more in case of ash particles which indicates complete stoppage of light radiations leading to further power reduction compared to soil and sand dust particles. It was also observed that the power output decreases with the increase in dust weight. Foreign particles on panel are mostly combination of these particulates so combination of these foreign particles was also considered during this study. At 30g dust and 700 W/m^2^ intensity, decrease in exergy output for case “E”, “F”, “G” and “H” was 43.20%, 49.47%, 60.70% and 50.18% respectively compared with clean panel. Hence, power output with mixture (ash as major constituent) resulted in lowest when it comes to particulate mixture case. It was observed that with the rise in intensity, the output power also increases. It can be seen that change in intensity from 500 to 700 W/m^2^ resulted in change in output power up to 36.5%. Same increasing power output trends were obtained with the dust on PV panel. At 30g and 700 W/m^2^, sand resulted in 44.8%, soil resulted in 91.2% and ash resulted in 71.1% enhancement in output power compared with output power at 500 W/m^2^. This rise in percentage means that greater intensity has less impact on output power compared to less intensity results. Similar trend was observed at 50g dust accumulation. Exergy output for case “E”, “F”, “G” and “H” was 61.64%, 79.42%, 79.23% and 70.53% respectively compared with clean panel. In all cases, increasing trend in exergy output was observed with the increase in light intensity. However, increase in ash as a major constituent resulted in lower power output compared to sand and soil as a major constituent. It was mainly due to more percentage of ash in the mixture which blocks most of the solar radiations. Moreover, with the increase in dust accumulation weight on solar panel resulted in the decline of power output. Thus, greater the quantity of foreign particulate, lesser will be the electrical output generated. At 50g and 700 W/m^2^, sand resulted in 52.4%, soil resulted in 79.8% and ash resulted in 63.4% enhancement in output power compared with output power at 500 W/m^2^. This rise in percentage means that greater intensity has less impact on output power compared to less intensity results. Similar results have been received as with 30g dust samples. Tables 4 & 5 represents the highest values recorded for each case in exergy output at 30g and 50g respectively. Furthermore, it was seen that PV output power with sand resulted in 9.7% enhancement when 50g dust was reduced to 30g. Similar rise in percentage was observed for other cases as well by reducing dust over PV surface.

**Table 4:** Exergy output at 30g dust accumulation.

Exergy Output (W) - 30g dust	500W/m^2^	600W/m^2^	700W/m^2^
**Clean Panel**	15.624	17.84	21.375
**Sand**	11.664	15.984	16.875
**Soil**	5.778	10.682	11.88
**Ash**	3.28	5.486	6.27
**Mixture (sand as main constituent)**	8.52	10.368	12.141
**Mixture (soil as main constituent)**	4.83	9.81	10.8
**Mixture (ash as main constituent)**	4.389	7.49	8.4
**Mixture (all constituents in equal quality)**	5.824	8.52	10.65

**Table 5:** Exergy output at 50g dust accumulation.

Exergy Output (W) - 50g dust	500W/m^2^	600W/m^2^	700W/m^2^
**Clean Panel**	15.624	17.84	21.375
**Sand**	10.011	14.52	15.4
**Soil**	4.06	4.761	5.075
**Ash**	0.9	1.9	2.88
**Mixture (sand as main constituent)**	3.6	6.816	8.2
**Mixture (soil as main constituent)**	2.388	4.08	4.4
**Mixture (ash as main constituent)**	1.692	3.762	4.439
**Mixture (all constituents in equal quality)**	3	4.738	6.3

**Fig 6:**
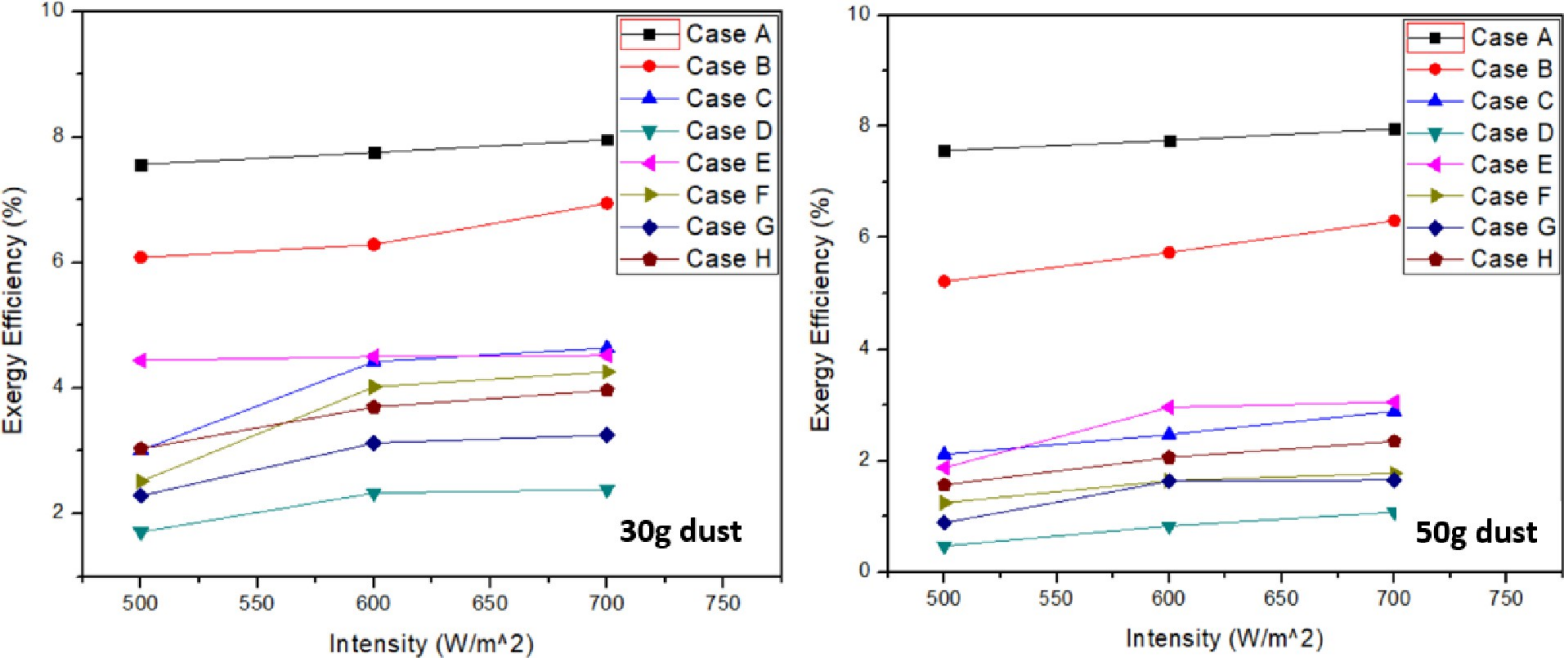
Exergy efficiency at 30g and 50g dust accumulation.

Following Fig 6 represents the exergy efficiency for all the cases at variable dust accumulation and intensity. Increase in light intensity resulted in exergy efficiency enhancement. Exergy efficiency was greater with 30g dust accumulation compared to 50g dust accumulation. Lowest exergy efficiency was observed for case “D” which is ash dust particle. Maximum exergy efficiency was observed for clean panel. Exergy efficiency was 12.76%, 41.70% and 70.06% was lower for sand, soil and ash respectively when compared with clean panel at 70 W/m^2^ and 30g dust. Similarly, 50g sand, soil and ash accumulation on solar panel resulted in 20.75%, 63.70% and 86.53% fall compared to clean panel exergy efficiency respectively. Same trends were received for exergy efficiency for case “E”, “F”, “G”, and “H”. For mixture containing all constituents in equal quantity, decrease in efficiency was 50.18% and 70.53% with 30g and 50g dust respectively compared with clean panel at an intensity of 70 W/m^2^. Tables 6 & 7 represents the highest values recorded for each case in exergy efficiency at 30g and 50g respectively. It can be depicted from the results that increase in intensity in ash and soil results in abrupt increase in power output. However, it is not the same in case of soil. It can also be observed in case of sand as major constituent in case E compared to other dust mixtures i.e., case F and case G. Furthermore, sand has less impact on power output when compared with all other dusts. Similar trends are observed when exergy efficiency was calculated for each case. In case E, 2.2% efficiency was increased when solar intensity was increased from 500–700 W/m^2^ and 30 g dust. But for other mixtures, change in efficiency was quite high especially in case of soil as a major constituent. It demonstrates that soil blocks more rays at lower intensity but at higher intensity, it declines resulting in abrupt change in outpower. Another mixture comprising of equal quantity of dust particles by weight was considered and it was concluded that its efficiency was better compared to ash and ash as a major constituent case. It was also confirmed form the results reduces the transmittance in greater extent compared to other dust particles, leading to low output power and efficiency. Thus, exergy efficiency for this mixture i.e., case H was 21.8% and 43.75% greater than ash as a major constituent in mixture case G at 30g and 50g dust respectively and 700 W/m^2^. However, it was lower than other mixtures with soil and sand as a major constituent.

**Table 6:** Exergy efficiency at 30g dust accumulation.

Exergy Efficiency (%) - 30g dust	500W/m^2^	600W/m^2^	700W/m^2^
**Clean Panel**	7.56425	7.75139	7.96057
**Sand**	6.08154	6.28466	6.94497
**Soil**	3.01261	4.4244	4.64127
**Ash**	1.71017	2.3351	2.38364
**Mixture (sand as main constituent)**	4.44228	4.50484	4.5216
**Mixture (soil as main constituent)**	2.51833	4.02218	4.26239
**Mixture (ash as main constituent)**	2.2884	3.12836	3.25437
**Mixture (all constituents in equal quality)**	3.0366	3.7019	3.96632

**Table 7:** Exergy efficiency at 50g dust accumulation.

Exergy Efficiency (%) - 50g dust	500W/m^2^	600W/m^2^	700W/m^2^
**Clean Panel**	7.56425	7.75139	7.96057
**Sand**	5.21967	5.73533	6.30887
**Soil**	2.11686	2.46863	2.89005
**Ash**	0.46925	0.82554	1.07258
**Mixture (sand as main constituent)**	1.87702	2.96152	3.05388
**Mixture (soil as main constituent)**	1.24509	1.63867	1.77274
**Mixture (ash as main constituent)**	0.8822	1.63457	1.65319
**Mixture (all constituents in equal quality)**	1.56418	2.05864	2.34627

### 3.2 Exergy losses

**Fig 7:**
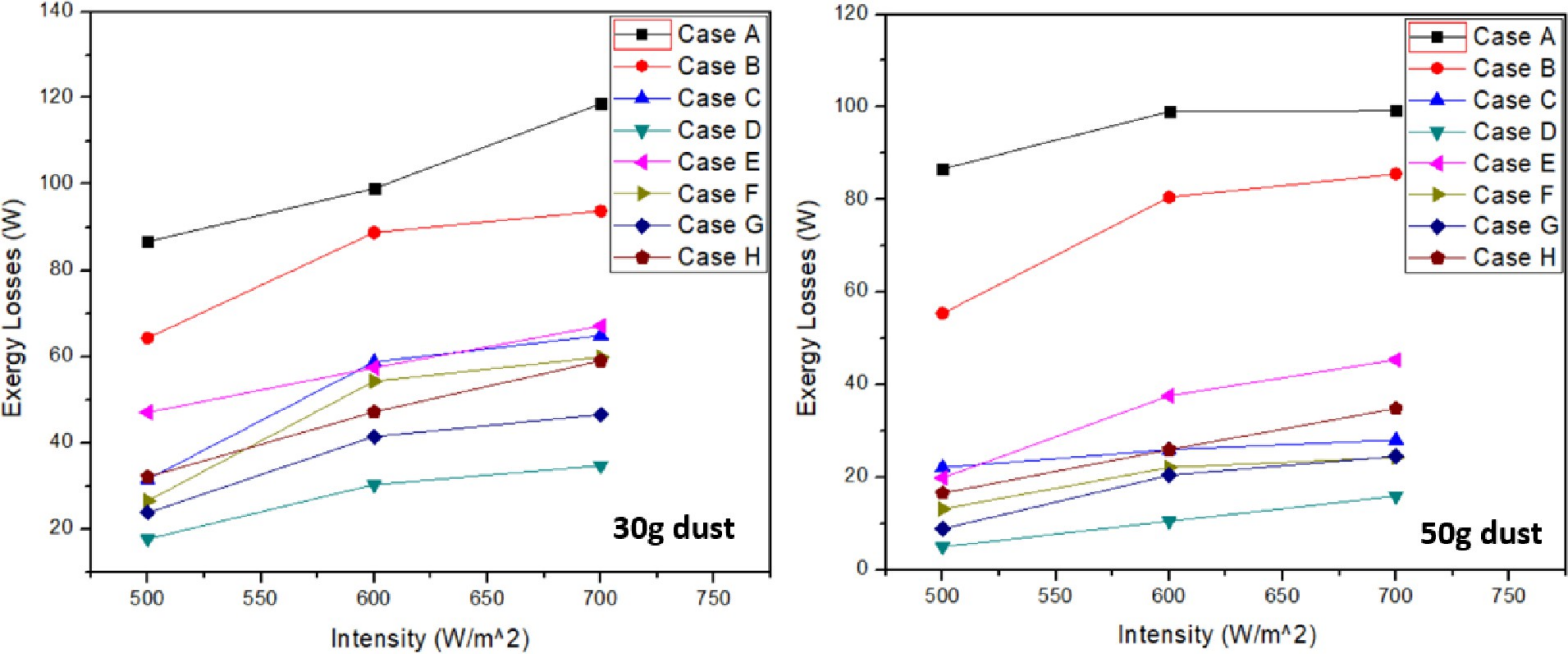
Exergy losses at 30g and 50g dust accumulation.

Exergy losses play an important role to increase overall performance of the system. It is equal to the sum of radiation exergy and convection exergy loss. Some of these are recoverable and some are unrecoverable. Fig 7 shows exergy loss for each case. Exergy loss for clean PV panel was more at all intensities considered during this experimentation due to higher temperature of panel. From Fig 7, it can be depicted that as intensity increases, exergy loss also increases. Maximum exergy loss observed was for clean panel and minimum exergy loss was for case “D” having ash particulate on solar panel. It was concluded that increase in dust accumulation on solar panel causes decrease in exergy loss. Furthermore, exergy losses were increased with the increase in light intensity. Increase in dust accumulation from 30g to 50g resulted in 8.78%, 56.81% and 54.15% for sand, soil and ash respectively at 700 W/m^2^. Similarly, mixture with dust accumulation resulted in 32.36%, 59.39%, 47.26% and 40.97% for case “E”, “F”, “G” and “H” respectively at same intensity. Tables 8 & 9 represents the highest values recorded for each case in exergy losses at 30g and 50g respectively. Graphs for exergy losses illustrates that with the increase in intensity, exergy losses also increase. Some of these losses can be recovered and overall efficiency of the system can be improved. Thus, in case of clean panel, the exergy losses were greater as compared to all other cases. Minimum losses were of ash dust particles case D in both 30g and 50g dust which indicates that recoverable losses are quite low for this case and exergy destruction is quite high for it. Thus, ash dust results in worst overall performance due to greater irreversibility involved in it. Soil also resulted in very low exergy losses compared to sand dust particle which can be seen in case C and case F both 30g and 50g.

**Table 8:** Exergy losses at 30g dust accumulation.

Exergy Losses (W) - 30g dust	500W/m^2^	600W/m^2^	700W/m^2^
**Clean Panel**	86.648	98.977	118.646
**Sand**	64.302	88.776	93.762
**Soil**	31.504	58.73	64.863
**Ash**	17.664	30.284	34.664
**Mixture (sand as main constituent)**	47.045	57.393	67.091
**Mixture (soil as main constituent)**	26.518	54.275	59.844
**Mixture (ash as main constituent)**	23.749	41.434	46.489
**Mixture (all constituents in equal quality)**	32.058	47.156	59.008

**Table 9:** Exergy losses at 50g dust accumulation.

Exergy Losses (W) - 50g dust	500W/m^2^	600W/m^2^	700W/m^2^
**Clean Panel**	86.648	98.977	118.646
**Sand**	55.389	80.494	85.529
**Soil**	22.089	25.964	28.013
**Ash**	4.962	10.481	15.895
**Mixture (sand as main constituent)**	19.876	37.604	45.378
**Mixture (soil as main constituent)**	13.132	22.089	24.303
**Mixture (ash as main constituent)**	8.825	20.429	24.519
**Mixture (all constituents in equal quality)**	16.559	25.964	34.830

At 700 W/m^2^, exergy losses reduces to 24.3 W, 53.2 W and 83.4 W for cases “B”, “C” and “D” respectively when compared with clean panel case A at 30g. In terms of mixtures, it reduces to 50.9 W, 58.2 W, 71.6 W and 59 W for cases “E”, “F”, “G” and “H” respectively at same intensity and 30g dust. Similarly, for 50g dust and 700 W/m^2^ intensity, exergy losses reduces to 32.5 W, 90 W and 102.1 W for cases “B”, “C” and “D” respectively when compared with clean panel case A. In terms of mixtures, it reduces to 72.6 W, 93.7 W, 93.5 W and 83.2 W for cases “E”, “F”, “G” and “H” respectively at same intensity and 50g dust.

### 3.3 Exergy destruction

**Fig 8:**
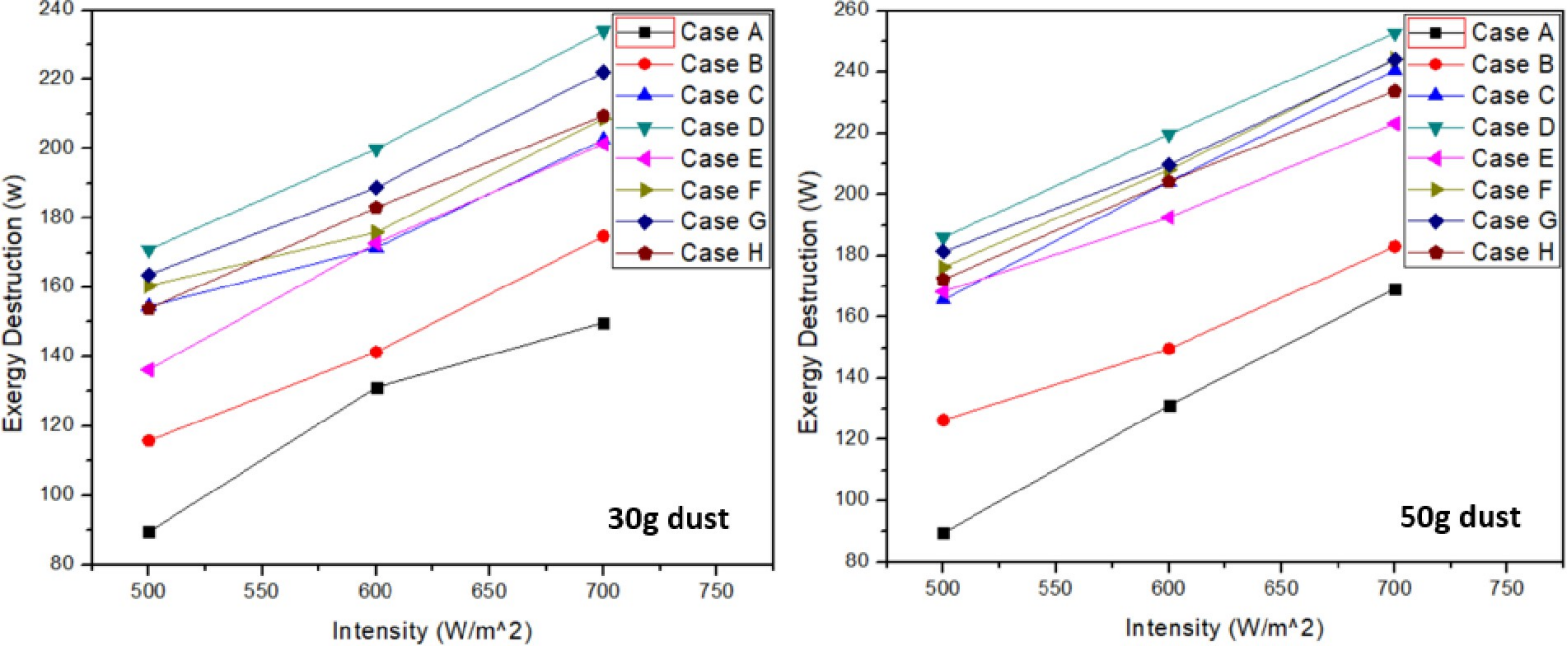
Exergy destruction at 30g and 50g dust accumulation.

Exergy destruction measures the non-utilizable energy due to some external or internal effect and goes wasted. In this case, exergy destruction was because of dust accumulation on PV panel surface which hinders the light impact on the PV cells. Fig 8 shows that for PV panel with ash accumulation, exergy destruction was highest at given intensities which reflects that most of the energy was wasted in case “D”. However, minimum exergy destruction was observed for case “A”. Thus, clean PV panel energy wastage is less as compared to all other cases. From Fig 8, it can be seen that sand accumulation on solar panel has much less energy wastage compared to other foreign particulates with case “C”, “D”, “E”, “F”, “G” and “H”. An increasing trend in exergy destruction was seen with the increase in light intensity and dust accumulation. Furthermore, it was concluded that the foreign particles accumulation hinders the solar panel from giving maximum power output. Therefore, cleaning of solar panel from time to time will result in better electrical output. By this way, energy can be saved from being wasted. Tables 10 &11 represents the highest values recorded for each case in exergy destruction at 30g and 50g respectively.

**Table 10:** Exergy destruction at 30g dust accumulation.

Exergy Destruction (W/K) - 30g dust	500W/m^2^	600W/m^2^	700W/m^2^
**Clean Panel**	89.52132	131.1757	149.8655
**Sand**	115.8275	141.3765	174.7492
**Soil**	154.5119	171.4228	202.6476
**Ash**	170.8494	199.8679	233.8471
**Mixture (sand as main constituent)**	136.2286	172.7564	201.4205
**Mixture (soil as main constituent)**	160.4461	175.8769	208.6675
**Mixture (ash as main constituent)**	163.6553	188.7183	222.0218
**Mixture (all constituents in equal quality)**	153.9116	182.9961	209.503

**Table 11:** Exergy destruction at 50g dust accumulation.

Exergy Destruction (W/K) - 50g dust	500W/m^2^	600W/m^2^	700W/m^2^
**Clean Panel**	89.52132	131.1757	149.8655
**Sand**	126.394	149.6581	182.9822
**Soil**	165.6445	204.1886	240.4981
**Ash**	185.9312	219.671	252.6158
**Mixture (sand as main constituent)**	168.3174	192.5483	223.1332
**Mixture (soil as main constituent)**	176.2732	208.0632	244.2082
**Mixture (ash as main constituent)**	181.2766	209.723	243.9923
**Mixture (all constituents in equal quality)**	172.2351	204.1886	233.6807

Exergy destruction results showed that with the increase in intensity, exergy destruction also increases. This shows the energy which goes wasted and overall efficiency of the system decreases. Thus, in case of clean panel, the exergy destruction is lowest as compared to all other cases. Maximum exergy is destroyed with ash dust particles case D in both 30g and 50g dust which indicates that waste of energy is quite high for this case. Thus, ash dust results in worst overall performance due to greater irreversibility involved in it.

At 700 W/m^2^, exergy destruction increases to 24.7 W, 52.6 W and 83.8 W for cases “B”, “C” and “D” respectively when compared with clean panel case A at 30g. In terms of mixtures, it increases to 50.9 W, 58.2 W, 71.6 W and 59 W for cases “E”, “F”, “G” and “H” respectively at same intensity and 30g dust. Similarly, for 50g dust and 700 W/m^2^ intensity, exergy destruction increases to 32.5 W, 90 W and 102.1 W for cases “B”, “C” and “D” respectively when compared with clean panel case A. In terms of mixtures, it increases to 72.6 W, 93.7 W, 93.5 W and 83.2 W for cases “E”, “F”, “G” and “H” respectively at same intensity and 50g dust.

The exergy destruction increases by 67.3%, 50.8%, 31.1% and 36.8% for cases “A”, “B”, “C” and “D” respectively at 30g dust when intensity increases from 500 to 700 W/m^2^. Similarly for mixtures, it was 47.8%, 30%, 35.5% and 36.1% for cases “E”, “F”, “G” and “H” respectively at 30g dust. Similar trend can be seen for 50g dust particles.

### 3.4 Entropy generation

**Fig 9:**
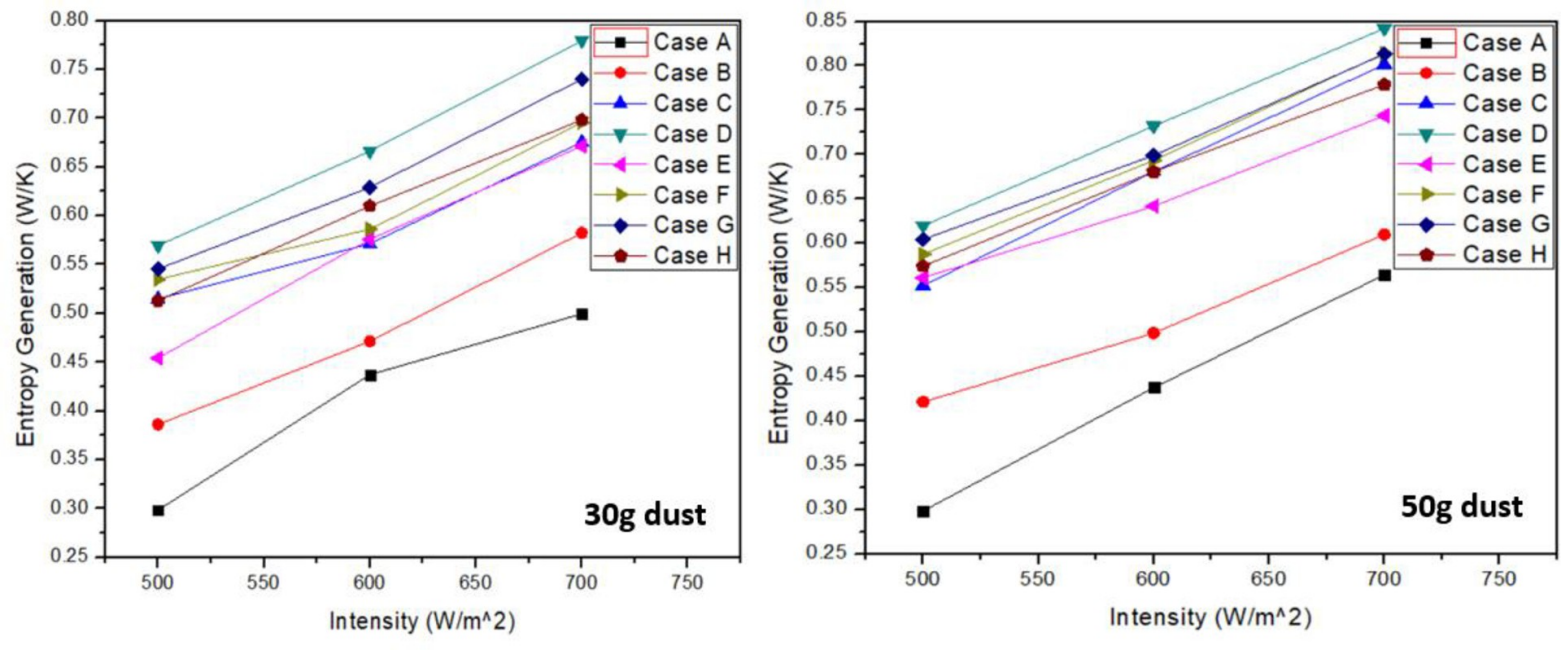
Entropy generation at 30g and 50g dust accumulation.

Disorderness of the system is measured by entropy generation. Fig 9 shows that for PV panel with ash accumulation, entropy generation was highest at given intensities. It showed the most disorder thus resulting into less useful work. However, minimum entropy generation was observed in case “A”. Thus, clean PV panel provided maximum useful work compared to all other cases. From Fig 9, it can be observed that increase in light intensity and dust accumulation resulted in higher entropy generation leading to an unstable system. With the increase in 20g dust accumulation, 4.71%, 18.68% and 8.03% increase in entropy generation was observed for sand, soil and ash respectively at 700 W/m^2^ intensity. Similarly, considering mixture constituents, increase in entropy generation was 10.78%, 17.03%, 9.90% and 11.54% for case “E”, “F”, “G” and “H” respectively. Tables 12 & 13 represents the highest values recorded for each case in entropy generation at 30g and 50g respectively.

**Table 12:** Entropy generation at 30g dust accumulation.

Exergy Generation (W/K)– 30g dust	500W/m^2^	600W/m^2^	700W/m^2^
**Clean Panel**	0.2984	0.43725	0.49955
**Sand**	0.38609	0.47125	0.5825
**Soil**	0.51504	0.57141	0.67549
**Ash**	0.5695	0.66623	0.77949
**Mixture (sand as main constituent)**	0.4541	0.57586	0.6714
**Mixture (soil as main constituent)**	0.53482	0.58626	0.69556
**Mixture (ash as main constituent)**	0.54552	0.62906	0.74007
**Mixture (all constituents in equal quality)**	0.51304	0.60999	0.69834

**Table 13:** Entropy generation at 30g dust accumulation.

Exergy Generation (W/K) - 50g dust	500W/m^2^	600W/m^2^	700W/m^2^
**Clean Panel**	0.2984	0.43725	0.49955
**Sand**	0.42131	0.49886	0.60994
**Soil**	0.55215	0.68063	0.80166
**Ash**	0.61977	0.73224	0.84205
**Mixture (sand as main constituent)**	0.56106	0.64183	0.74378
**Mixture (soil as main constituent)**	0.58758	0.69354	0.81403
**Mixture (ash as main constituent)**	0.60425	0.69908	0.81331
**Mixture (all constituents in equal quality)**	0.57412	0.68063	0.77894

Entropy generation results showed that with the increase in intensity, entropy generation also increases. In case of ash dust panel, the entropy generation is maximum which reflects that the disorderness in the system is more when ash dust is over the PV surface compared to soil and sand dust particles. However, for clean panel, entropy generation is lowest as compared to all other cases in both 30g and 50g dust. Thus, ash dust results in worst overall performance due to greater disorder generated leading to lower useful work.

Entropy generation increases to 3.57%, 18.8% and 8.08% for cases “B”, “C” and “D” respectively when dust weight was increased form 30g to 50g at 700 W/m^2^. In terms of mixtures, it increases to 10.7%, 17.1%, 9.8% and 11.6% for cases “E”, “F”, “G” and “H” respectively at same intensity and dust weight increased from 30g to 50g.

### 3.5 Sustainability index

**Fig 10:**
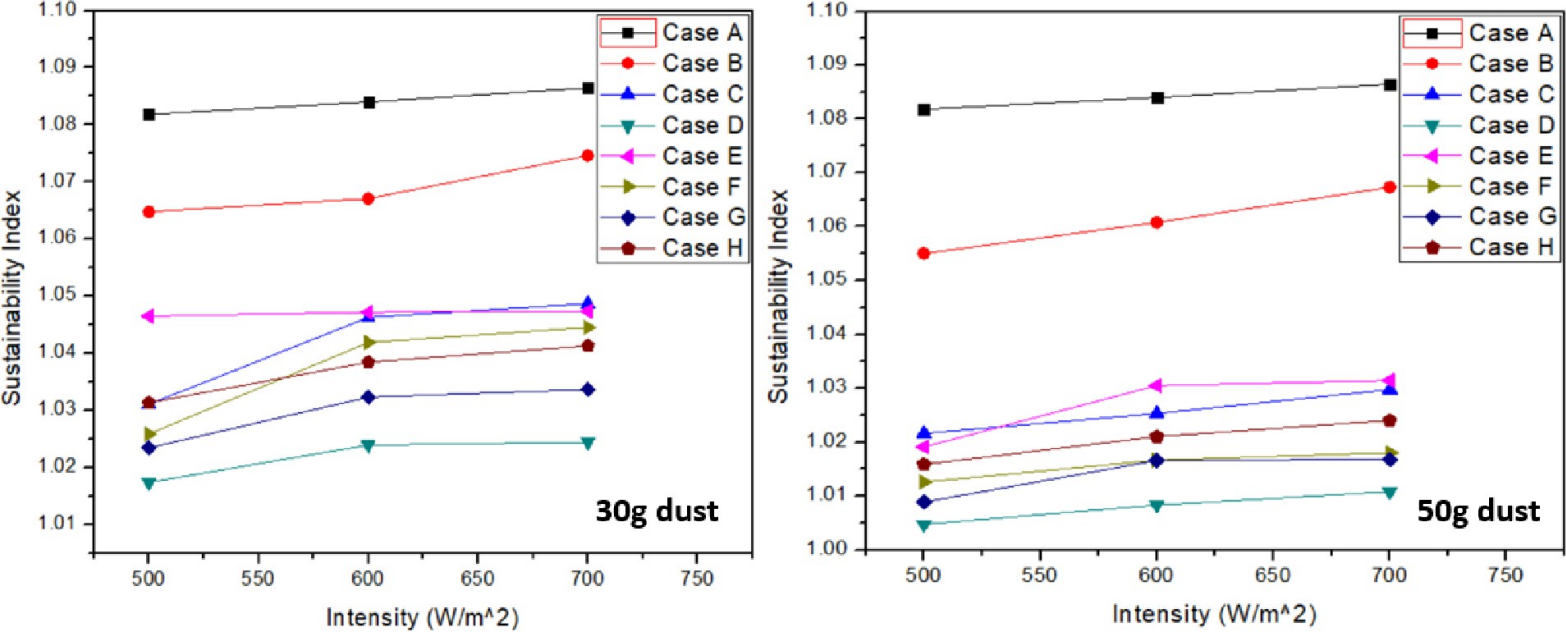
Sustainability index at 30g and 50g dust accumulation.

Sustainability index shows the performance of system based on the exergy output. From Fig 10, it is clearly predicted that clean panel case “A” represents maximum sustainability index of 1.086. However, minimum sustainability index value was for case “D” with 1.016 at 500 W/m^2^ intensity and 50g dust accumulation. It was concluded that sustainability index was increased with the increase in light intensity. However, increase in dust accumulation resulted in the decline of sustainability index. With the addition of 30 g sand, soil and ash reduces sustainability index by 1.09%, 3.48% and 5.71% respectively at 700 W/m^2^ when compared with clean panel. At 50g dust accumulation, sustainability index reduces further to 1.76%, 5.22% and 6.96% by addition of sand, soil and ash respectively compared with clean panel at same intensity. This indicates that increase in dust accumulation directly effects the performance output of solar panel. Tables 14 & 15 represents the highest values recorded for each case in sustainability index at 30g and 50g respectively.

**Table 14:** Sustainability index at 30g dust accumulation.

Sustainability Index - 30g dust	500W/m^2^	600W/m^2^	700W/m^2^
**Clean Panel**	1.08183	1.08403	1.08649
**Sand**	1.06475	1.06706	1.07463
**Soil**	1.03106	1.04629	1.04867
**Ash**	1.0174	1.02391	1.02442
**Mixture (sand as main constituent)**	1.04649	1.04717	1.04736
**Mixture (soil as main constituent)**	1.02583	1.04191	1.04452
**Mixture (ash as main constituent)**	1.02342	1.03229	1.03364
**Mixture (all constituents in equal quality)**	1.03132	1.03844	1.0413

**Table 15:** Sustainability index at 50g dust accumulation.

Sustainability Index - 50g dust	500W/m^2^	600W/m^2^	700W/m^2^
**Clean Panel**	1.08183	1.08403	1.08649
**Sand**	1.05507	1.06084	1.06734
**Soil**	1.02163	1.02531	1.02976
**Ash**	1.00471	1.00832	1.01084
**Mixture (sand as main constituent)**	1.01913	1.03052	1.0315
**Mixture (soil as main constituent)**	1.01261	1.01666	1.01805
**Mixture (ash as main constituent)**	1.0089	1.01662	1.01681
**Mixture (all constituents in equal quality)**	1.01589	1.02102	1.02403

It can be seen that with the increase in intensity, sustainability index also increases. With the increase in dust weight, sustainability index decreases. At 700 W/m^2^ intensity, sustainability index decreases by 0.6%, 1.8% and 1.3% for cases “B”, “C” and “D” respectively when dust weight was increased form 30g to 50g. In terms of mixtures, it decreases by 1.5%, 2.4%, 1.6% and 1.6% for cases “E”, “F”, “G” and “H” respectively at same intensity.

## 4. Conclusion

An indoor experimentation was performed on PV panels to extract the actual utilizable energy under various circumstances caused due to atmospheric high dust particle ratio predicted at Peshawar, Pakistan. Based on the current scenario, effect of dust accumulation on PV panels installed at the roof top is considered in this study. Dirt in this city is usually comprised of sand, soil and ash with varying concentration ratio. Following results were concluded after the experimentation:

1. Maximum exergy output and exergy efficiency was recorded for case A i.e., clean PV panel which indicated that dirt accumulation has a direct effect on the output of PV panel
2. Minimum exergy output was achieved at 50g case D i.e., ash accumulation which clearly states that accumulation of ash has greater effect on solar PV panel
3. Impact of ash was further analyzed by using multiple constituents of mixture in which case G i.e., mixture (ash as main constituent) accumulation gives lowest PV panel efficiency and output as it decreases PV output drastically
4. Maximum exergy destruction was during ash accumulation in case D. The impact of soil accumulation was more than sand accumulation due to higher exergy destruction. Furthermore, exergy destruction for 50g dust accumulation was more when compared to 30g dust accumulation
5. Increase in intensity in both 30g and 50g dust accumulation resulted in an increase in exergy output, efficiency and more exergy losses
6. Exergy losses and sustainability index were higher for clean panel and lowest for ash accumulation on PV panel whereas entropy generation showed similar trends like exergy destruction for all cases

Thus, it was seen that dust accumulation results in higher losses and less output therefore, use of solar PV panels requires cleaning to eliminate the decline in daily power output. Clean panels give maximum output which can be identified from the results in this study. In future studies, impact of air humidity and wind speed on dust accumulation and power output will be studied. In addition to this, effect of rain over dust accumulated PV surface will be considered and real time experimentation will be conducted at mining sites and industries.

## Abbreviations

*Parameters* A: surface area (m^2^)
Cp: specific heat (J kg^-1^K^-1^)
G: solar irradiation (W m^-2^)
k: thermal conductivity (W/m.K)
m: mass (g)
PV: photovoltaic
T: temperature (K)
SI: sustainability index
IP: improvement potential
*Subscripts* amb: Ambient
bf: base fluid
nf: nanofluid
elect: electrical
dest: destruction
*Greeks* p: density (kg m^-3^)
η: efficiency
Ėn: energy
Ėx: exergy
$\dot{S}$: entropy
